# Cationic hydrogel with sustained lubrication and anti-calcification for osteoarthritis therapy

**DOI:** 10.1186/s12951-026-04164-0

**Published:** 2026-03-01

**Authors:** Longying Sun, Zhenxing Guo, Wenpin Qin, Xiaokang Zhang, Dongxiao Hao, Weiwei Zhu, Xiaoxiao Han, Yuxuan Hou, Le Mao, Weicheng Lu, Yao Zhao, Kai Jiao

**Affiliations:** 1 Department of Stomatology, State Key Laboratory of Oral and Maxillofacial Reconstruction and Regeneration & School of Stomatology, Tangdu Hospital, The Fourth Military Medical University, Xi’an, Shaanxi China; 2 State Key Laboratory of Oral and Maxillofacial Reconstruction and Regeneration & National Clinical Research Center for Oral Diseases & Shaanxi Key Laboratory of Stomatology, School of Stomatology, The Fourth Military Medical University, Xi’an, Shaanxi China; 3 Shaanxi University of Chinese Medicine, No.1, Shiji Avenue, Xianyang, Shaanxi China; 4 Department of Stomatology, Lanzhou University, Lanzhou University, Lanzhou, China; 5 College of Chemistry, Fuzhou University, Fuzhou, China

**Keywords:** Osteoarthritis, Alkylated chitosan, Cationic hydrogel, Lubrication, Pathological calcification

## Abstract

**Background:**

Osteoarthritis is characterized by cartilage degradation and abnormal subchondral bone remodeling, primarily due to disrupted lubrication–calcification coupling. Current treatments mainly relieve symptoms but lack effective strategies against calcification, and lubrication supplements offer only temporary relief. Here, we present a novel dual-functional cationic hydrogel designed to simultaneously enhance lubrication and inhibit pathological calcification, targeting the dysregulated lubrication–calcification interaction in OA. This approach offers a promising disease-modifying strategy for OA treatment.

**Results:**

Formulated from alkylated modified chitosan (mCS) and lysine (Lys), the mCS/Lys hydrogel exhibits dual functionalities: inhibiting pathological calcification and enhancing joint lubrication. These effects are achieved through structural modifications that create a brush-like architecture, recruiting endogenous lubricating factors and calcification precursors due to its intrinsic surface activity. Compared to clinically utilized hyaluronic acid, mCS/Lys hydrogel demonstrates an extended duration of action and enhanced therapeutic efficacy. The intra-articular delivery of the hydrogel in a murine osteoarthritis model demonstrated that the mCS/Lys hydrogel effectively inhibits pathological calcification, alleviates pain, and promotes the repair of articular cartilage.

**Conclusions:**

Overall, the mCS/Lys hydrogel offers a more holistic and enduring treatment approach by integrating sustained lubrication with anti-calcification features. Compared to traditional single-target methods, it indicates a promising, translatable solution.

**Graphical abstract:**

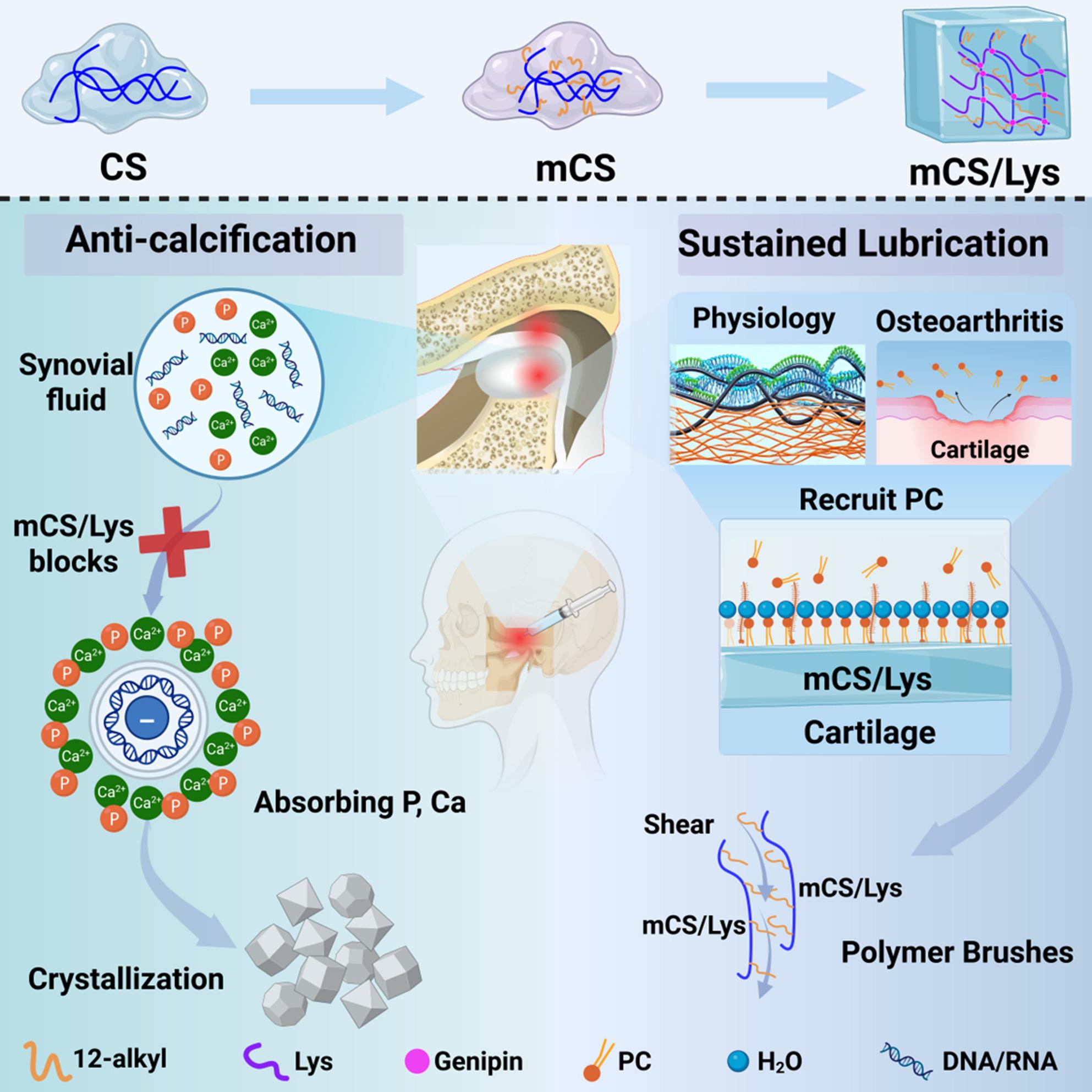

**Supplementary Information:**

The online version contains supplementary material available at 10.1186/s12951-026-04164-0.

## Background

Osteoarthritis (OA) is a debilitating joint disease with a rapidly rising global incidence, affecting over 595 million people by 2021 and projected to reach 1.1016 billion by 2050 [1]. Key features include cartilage degradation, abnormal subchondral bone remodeling, and synovial inflammation [2]. A crucial but often overlooked factor in OA progression is the “lubrication-calcification coupling disruption” [3, 4]. Under normal conditions, boundary lubricants like phosphatidylcholine (PC) ensure low friction in joints [5]. The loss of these lubricants and cartilage damage increases friction and raises the coefficient of friction (COF) from normal levels (0.001–0.05) [5] to pathological levels (0.3–0.5) [6]. Additionally, calcification occurs when extracellular nucleic acids from dying chondrocytes bind calcium and phosphate, leading to mineral deposits that further damage the extracellular matrix [7, 8]. This creates a cycle of lubrication failure and calcification, worsening joint dysfunction. Modulating the aberrant lubrication-calcification coupling in OA cartilage could be a key approach to mitigate OA disease progression.

Current treatments for OA are primarily palliative, concentrating on alleviating symptoms rather than modifying the disease itself [9]. For instance, hyaluronic acid (HA) enhances joint lubrication but is characterized by rapid degradation, necessitating frequent administration [10, 11]. It also fails to inhibit calcification [12]. Although novel approaches, such as liposomes [13–15] and self-lubricating hydrogels [16, 17], demonstrate improved lubrication, they are hindered by complex synthesis processes, concerns regarding biocompatibility, and limited functionality [18, 19]. Similar to HA, these lubricants necessitate frequent administration, and the lubrication performance is unable to sustain effectiveness over prolonged friction. Importantly, none of these strategies addresses the interdependent relationship between impaired lubrication and pathological calcification, both of which are central to OA pathogenesis [20]. Therefore, there is a pressing need for sustained therapies that concurrently target lubrication-calcification coupling to effectively disrupt the OA disease cycle.

Chitosan (CS), a naturally occurring cationic polysaccharide approved by the FDA for its excellent biocompatibility, can be chemically modified through alkylation to produce alkylated chitosan (mCS) [21]. This modification preserves its cationic charge, facilitating anion adsorption. Besides, it enhances its bioactivity and structural stability [22], thereby improving its lubricating properties [23]. Lysine (Lys), an essential cationic amino acid, is known to inhibit pathological calcification [24] and bind to lubricants such as PC [25]. Both mCS and Lys utilize their positive charges to electrostatically capture anionic lubricants, such as PC, and calcification precursors, including extracellular nucleic acids, thereby maintaining a dynamic equilibrium [26, 27]. The cationic properties of these substances enhance adhesion to the cartilage matrix through electrostatic and hydrophobic interactions [28, 29]. While these components potentially offer a dual function of lubrication and calcification inhibition, it remains uncertain whether their combined application can effectively suppress the progression of OA.

**Scheme 1:**
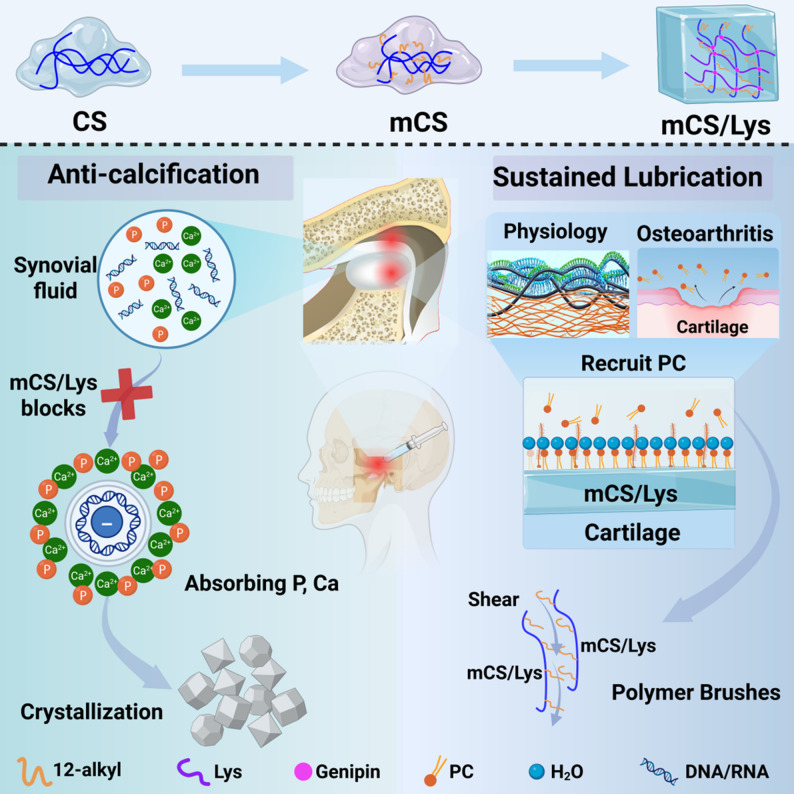
Synthesis of an injectable cationic mCS/Lys hydrogel and its dual action in OA therapy: long-term lubrication and suppression of pathological calcification. chitosan (CS); alkylated chitosan (mCS); lysine (Lys); phosphatidylcholine (PC)

To address this, we developed an injectable dual‑functional cationic mCS‑Lys hydrogel through a chemical modification process (Scheme 1). The hydrogel exhibited excellent injectability and biocompatibility; its unique brush‑like molecular architecture and electrostatic interactions significantly lowered the COF of cartilage synovial fluid compared with HA, and provided long‑lasting lubrication for over 35 days. Moreover, electrostatic adsorption of extracellular nucleic acids effectively reduced pathological calcification in osteoarthritis. Evaluation in a murine model of temporomandibular joint osteoarthritis (TMJOA) further demonstrated that, by modulating the aberrant lubrication–calcification coupling in OA, the hydrogel markedly slowed cartilage degeneration and alleviated pain, significantly delaying OA progression. These findings suggest a promising, translatable therapeutic strategy for OA treatment.

## Methods

### Materials

All chemicals were commercially sourced and used as received. Chitosan, Acetic Acid, L-lysine, Genipin, Sodium Hydroxide, 1,2-dimyristoyl-sn-glycero-3-phosphocholine (DMPC), and Dimethyl Sulfoxide were purchased from Macklin (China). Lauryl Aldehyde, Sodium Cyanoborohydride, and Fluorescein Isothiocyanate (FITC) came from Aladdin (China). Dexamethasone, L-ascorbic Acid, β-glycerophosphate Disodium Salt, Alizarin Red S, and Triton X-100 were obtained from MilliporeSigma (USA). Penicillin-streptomycin solution and Fetal Bovine Serum (FBS) were from Procell (China). Hyaluronidase, Safranin O/Fast green Staining solution, H&E Staining solution, and Alizarin Red S solution (1%, pH 4.2) were from Solarbio (China). Runx2 and SOX9 antibodies were from Santa Cruz (USA), cat: sc-390,351, sc-166,505. Minimum Essential Medium α (αMEM) and Phosphate Buffer Saline (PBS) werefrom Carlsbad (USA). LIVE/DEAD™ Cell Staining was from Invitrogen (USA). Hyaluronic Acid and Cell Counting Kit-8 (CCK-8) were from GLPBIO Technology LLC (USA).

### Synthesis of mCS/Lys hydrogels

#### Synthesis of mCS

CS (4 g) was dissolved in 200 mL of 2% acetic acid. After full dissolution, lauraldehyde was added at set molar ratios and stirred at 37 °C for 12 h. The pH was adjusted to 5.0 with 1 M NaOH, then sodium cyanoborohydride was added. After 3 h, the pH was neutralized to 7.0 with NaOH. The precipitate was washed with ethanol and freeze-dried to produce modified chitosan with different grafting levels.

#### Synthesis of mCS/Lys hydrogel

mCS (2 g) were dissolved in 200 mL of 2% acetic acid. Lys was added in a 1:1 molar ratio to mCS, and the mixture was stirred at 37 °C for four hours. Genipin was added for crosslinking, followed by sonication at 40 kHz for 30 min and dialysis against deionized water. After treating the hydrogel by neutralizing acetic acid with sodium hydroxide and performing dialysis, the pH was adjusted to a neutral to weakly alkaline range (7.3–7.8), matching the normal joint cavity’s pH [30, 31]. The resulting mCS/Lys hydrogels were stored at 4 °C [32–34].

Physicochemical analysis and molecular simulations suggest an optimal alkylation concentration of 10:1 to 10:3. For cytotoxicity and application purposes, a genipin to primary amines molar ratio of 1:1 to 1.5:1 is recommended. The hydrogel in this study was synthesized using alkyl chitosan with 10% amino substitution, with a final molar ratio of mCS: Lys: genipin at 4:4:3 [35, 36].

### Fundamental characterization

#### Structural and thermal analysis

The chemical structures were characterized using attenuated total reflectance-Fourier transform infrared spectroscopy (ATR-FTIR; model FTIR-8400 S, Shimadzu, Japan) within the spectral range of 4000–400 cm⁻¹, employing 32 scans at a resolution of 4 cm⁻¹, and analyzed using IRsolution software version 1.40 (Shimadzu). Thermogravimetric analysis (TGA) and differential scanning calorimetry (DSC) were conducted utilizing an STA 8000 instrument (PerkinElmer, USA) under a nitrogen atmosphere, with a heating rate of 10 °C/min over a temperature range of 35–800 °C. The degree of crystallinity was evaluated through X-ray diffraction (XRD) using an Empyrean diffractometer (Panalytical, Netherlands) with Cu Kα radiation (λ = 1.5406 Å), covering a scan range of 5°–50° with a step size of 0.02° and a scan speed of 2°/min. Data analysis was performed using Jade 9.0 software (MDI, USA) in conjunction with the ICDD PDF-4 + database.

#### Morphological and physicochemical analysis

Morphology was examined by scanning electron microscopy (SEM; S-4800, Hitachi, Japan) at 5 kV after gold sputter-coating (5 nm) of lyophilized hydrogels. ImageJ was used to quantify the porosity of hydrogels in SEM images. Rheological properties (viscoelasticity) were measured on a rheometer (Kinexus Lab+, NETZSCH, Germany) at 25 °C: frequency sweep (0.1–1000 Hz, 2% fixed strain) and shear-thinning test (shear rate 0–100 s⁻¹, 10 rad/s frequency, 1% strain).

#### Water-related properties

The water content of the hydrogels was assessed by immersing them in PBS containing 0.1 U/mL hyaluronidase at 37 °C. Following equilibration to a swollen state, the hydrogels were subjected to freeze-drying, and the water content percentage was determined using the formula [(M₁−M₂)/M₁] × 100, where M₁ represents the swollen mass, and M₂ denotes the dry mass. The residual mass percentage was calculated as (Mₜ/M₀) × 100, with M₀ indicating the initial mass and Mₜ the mass at a given time t [37]. Surface wettability was evaluated by measuring the water contact angle using an Easy Drop K100 instrument (Krüss, Germany) with 2 µL droplets applied to hydrogel surfaces measuring 10 mm × 2 mm (*n* = 5). Additionally, the zeta potential and hydrodynamic diameter were determined through dynamic light scattering (DLS) and electrophoretic light scattering (ELS) using a Litesizer 500 (Anton Paar, Austria) at 25 °C, ensuring a transmittance greater than 80% [26, 38–40].

#### Mechanical properties

Compressive modulus was measured by bio-nano indenter (Piuma, Optics11, Netherlands) using a spherical tip (radius 10 μm, 5 μm displacement) with force-indentation curves fitted to the Hertzian model (*n* = 5 samples, 80–100 indents/sample) [41]. Atomic force microscopy (AFM; Keysight 5500, USA) with a conical tip (force constant 13 N/m) was used for nanomechanical indentation (50 μm-thick samples) [42, 43].

#### Lubrication properties

The friction coefficients were assessed using a ball-on-disk tribometer (UMT-2MT, CETR, USA) with a silicon nitride ball (8 mm diameter) against Poly(2-Hydroxyethyl Methacrylate) (PHEMA) hydrogels, with an amplitude of 4 mm, a speed of 6 mm/s, at 37 °C, and 40–50% relative humidity; the lubricants used were either pure water or DMPC at 1 mg/mL [44]. Surface roughness was analyzed by 3D optical profilometry (ST 400, Nanovea, USA) with Nanovea Software v7.455. Simulated Synovial Fluid (SSF): PBS + 1% bovine serum albumin + 0.1% PC [45].

### Molecular dynamics (MD) simulations

The structures of mCS, HA, Lys, and DMPC were modeled using Charmm-gui, while extracellular nucleic acids were modeled with Discovery Studio. The FF19SB, OL24, GAFF2, and lipids21 force fields were applied to Lys, DNA, CS, HA, and DPPC, respectively. The solvated system underwent energy minimization using 5000 steps each of steepest descent and conjugate gradient algorithms, followed by heating from 0 K to 300 K over 500 ps. It was then relaxed with restrained MD simulation in the NVT and NPT ensembles for 500 ps each until equilibrium was achieved. A 100 ns MD simulation was conducted in the NPT ensemble, and the binding free energy was calculated using the MM-GBSA method.

### Biocompatibility

#### In vitro cytocompatibility

Cytotoxicity was assessed via CCK-8 assay with ATDC5 chondrocytes (5 × 10³ cells/well, 12/24 h incubation with hydrogel extracts; absorbance at 450 nm). Cell viability was visualized by live/dead staining (calcein-AM/propidium iodide) using confocal microscopy (A1R, Nikon, Japan). Hemolysis was quantified by incubating mouse RBCs with hydrogel extracts (1 h, 37 °C) and calculating hemolysis (%) from absorbance (540 nm) relative to Triton X-100 (positive control) and PBS (negative control).

#### In vivo biocompatibility

Hydrogels (10 µL) were intraperitoneally injected into C57BL/6J mice (*n* = 5). After 4 weeks, major organs such as the heart, liver, spleen, lung, and kidney were extracted, fixed in formalin, embedded in paraffin, sectioned, and stained with hematoxylin and eosin (H&E). Histological images were then obtained using light microscopy (Olympus, Japan).

### In vitro experiments

#### Cell culture and calcification induction

ATDC5 cells (CL-0856, Wuhan Pricella Biotechnology Co., Ltd) were grown in αMEM containing 10% FBS and 1% penicillin/streptomycin at 37 °C with 5% CO₂. The calcification medium included αMEM with 10 nM dexamethasone, 100 µM ascorbic acid, 1.1 mM CaCl₂, and 10 mM β-glycerophosphate.

#### Alizarin red S staining

After fixing the cells in 10% formaldehyde, they were stained with Alizarin Red S (40 mM, pH 4.2) for 30 min and rinsed. The mineralized nodules were quantified using ImageJ (NIH, USA) following destaining with cetylpyridinium chloride.

### Animal experiments

#### OA mice model and hydrogel administration

TMJOA was induced in C57BL/6J mice (8-week-old) via unilateral anterior crossbite (UAC) [46]. Hydrogels (10 µL) or PBS were injected into TMJ cavities. The animal protocols were approved by the Institutional Animal Care and Use Committee of the Fourth Military Medical University (IACUC-20250164). The positive control was an intra-articular injection of hyaluronic acid, while the negative control was an injection of PBS.

#### In vivo retention

FITC-labeled mCS/Lys hydrogels were injected into TMJ cavities, and fluorescence retention was monitored weekly for 5 weeks using IVIS (Lumina XRMS, PerkinElmer, USA; excitation/emission: 465/520 nm).

#### Behavioral and imaging assessments

The Von Frey test for mechanical allodynia thresholds, the open field test (OFT) for locomotor activity, and the elevated plus maze (EPM) for anxiety-like behaviors were conducted to assess pain behavior, with data analyzed using VisuTrack 8.0 from Softmaze, China.

#### MicroCT

Mandibles were scanned using a Quantum GX2 (PerkinElmer; 16 μm resolution) and reconstructed with Mimics 21.0 (Materialise, Belgium).

#### Histology

Sections of decalcified tissues (7 μm) were stained with H&E or Safranin O/Fast Green and examined using light microscopy (DM4, Leica, Germany). RUNX2 (F-2) is for detection of RUNX2 of mouse origin by immunofluorescence (starting dilution 1:50, dilution range 1:50 − 1:500), and Sox-9 (E-9) is for detection of Sox-9 of mouse origin by immunofluorescence (starting dilution 1:50, dilution range 1:50 − 1:500). ImageJ was used to quantify immunofluorescence for SOX9/RUNX2.

### Statistical analysis

Experiments were conducted with 3–5 replicates. Data normality and homoscedasticity were checked using the Shapiro-Wilk and Levene’s tests. Intergroup differences were analyzed with Student’s t-test for two groups and one-way ANOVA with Tukey’s post hoc test for multiple groups. The Kruskal-Wallis test was used for non-continuous variables like OARSI scores and some animal behavior assessments. Results are presented as mean ± standard deviation (SD). Statistical significance was determined according to the guidelines of the New England Journal of Medicine (NEJM). All analyses were conducted using GraphPad Prism version 9.5 (GraphPad Software, USA) and Origin 2024 (OriginLab Corporation, USA).

## Results and discussion

### Synthesis and characterization of the mCS/Lys hydrogel

**Fig. 1:**
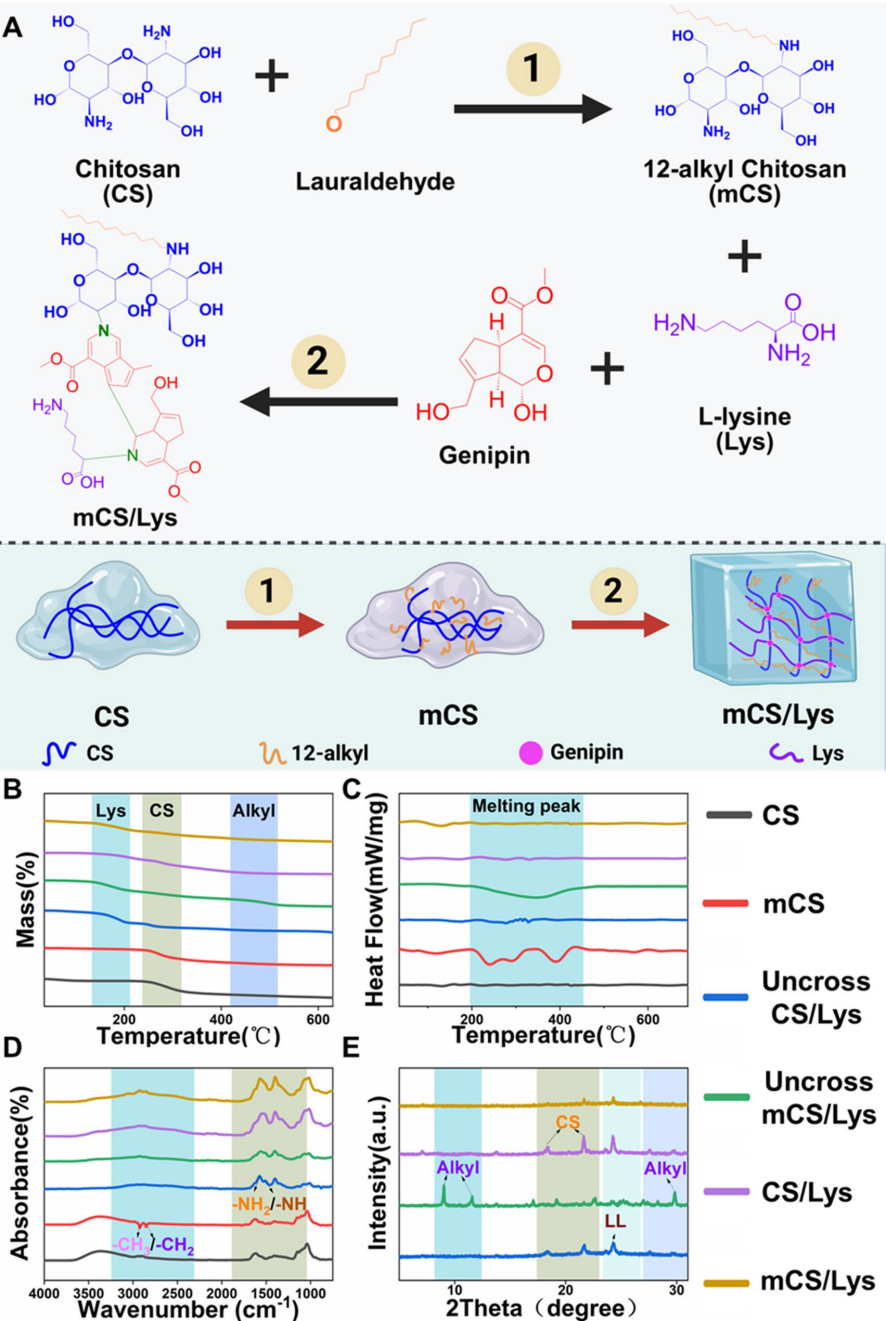
Synthesis and physicochemical characterization of mCS/Lys hydrogels. **A**) Synthetic scheme of mCS/Lys hydrogels. **B, C**) Thermodynamic parameters monitored throughout the crosslinking process: TGA and DSC. **D, E**) Spectroscopic confirmation of chemical structure: ATR-FTIR and XRD

To prepare the bifunctional cationic hydrogel, it was synthesized via a two-step method involving chemical modification and crosslinking. (Fig. 1A). In the first step of the synthesis, dodecyl side chains were introduced onto the chitosan backbone via reductive amination, yielding mCS [47]. Short-chain alkyl groups (C1–C3) lack sufficient hydrophobicity for stable nanostructures, while long-chain groups (C14+) are too hydrophobic, affecting water solubility and biocompatibility. Medium-to-long-chain alkyl groups (C6–C12) offer a balance, enabling effective self-assembly into nanoparticles and influencing properties like surface charge and drug release [48, 49]. These chains don’t significantly increase chitosan’s cytotoxicity and maintain its biodegradability [50]. For lubrication, C8 to C18 chains are common, with dodecyl (C12) being ideal for stability, fluidity, and low friction [51–53]. Dodecyl chitosan is well-researched, and its precursor, dodecyl aldehyde, is naturally occurring, supporting green chemistry principles [47, 54]. Hence, the dodecyl chain was chosen. Subsequently, a dual-crosslinked hydrogel network was constructed through genipin crosslinking in the presence of lysine [35, 54], effectively transforming the fluid mCS/lysine precursors into a mechanically stable hydrogel. Multiple batches of mCS/Lys hydrogels were successfully synthesized, each demonstrating high consistency with minimal variation in key performance metrics, thus indicating excellent reproducibility.

Thermodynamic analysis revealed the appearance of new alkyl-related decomposition peaks in the final product, along with an overall enhancement in thermal stability compared to pristine chitosan. These observations provide strong evidence for the successful alkylation of chitosan and its subsequent crosslinking with lysine (Fig. 1B–C). FTIR spectroscopy further supported the occurrence of amidation reactions. Specifically, a notable decrease in the intensity of the primary amine band near 1590 cm⁻¹, coupled with the emergence of a secondary amine signal in the 1540–1560 cm⁻¹ region, indicated the conversion of free amino groups into amide linkages (Fig. 1D). These spectral features were absent in both unmodified chitosan and lysine monomers, as shown in Fig. S2. XRD analysis showed a marked reduction in crystallinity, accompanied by the appearance of new reflections characteristic of alkyl chain incorporation, thereby confirming the structural modifications induced by alkylation and crosslinking (Fig. 1E). Collectively, the integrated spectroscopic, thermal, and morphological analyses unequivocally demonstrate the successful alkylation of chitosan and its effective crosslinking with lysine, laying a robust foundation for the synthesis of the dual-functional cationic hydrogel with tailored physicochemical properties.

**Fig. 2:**
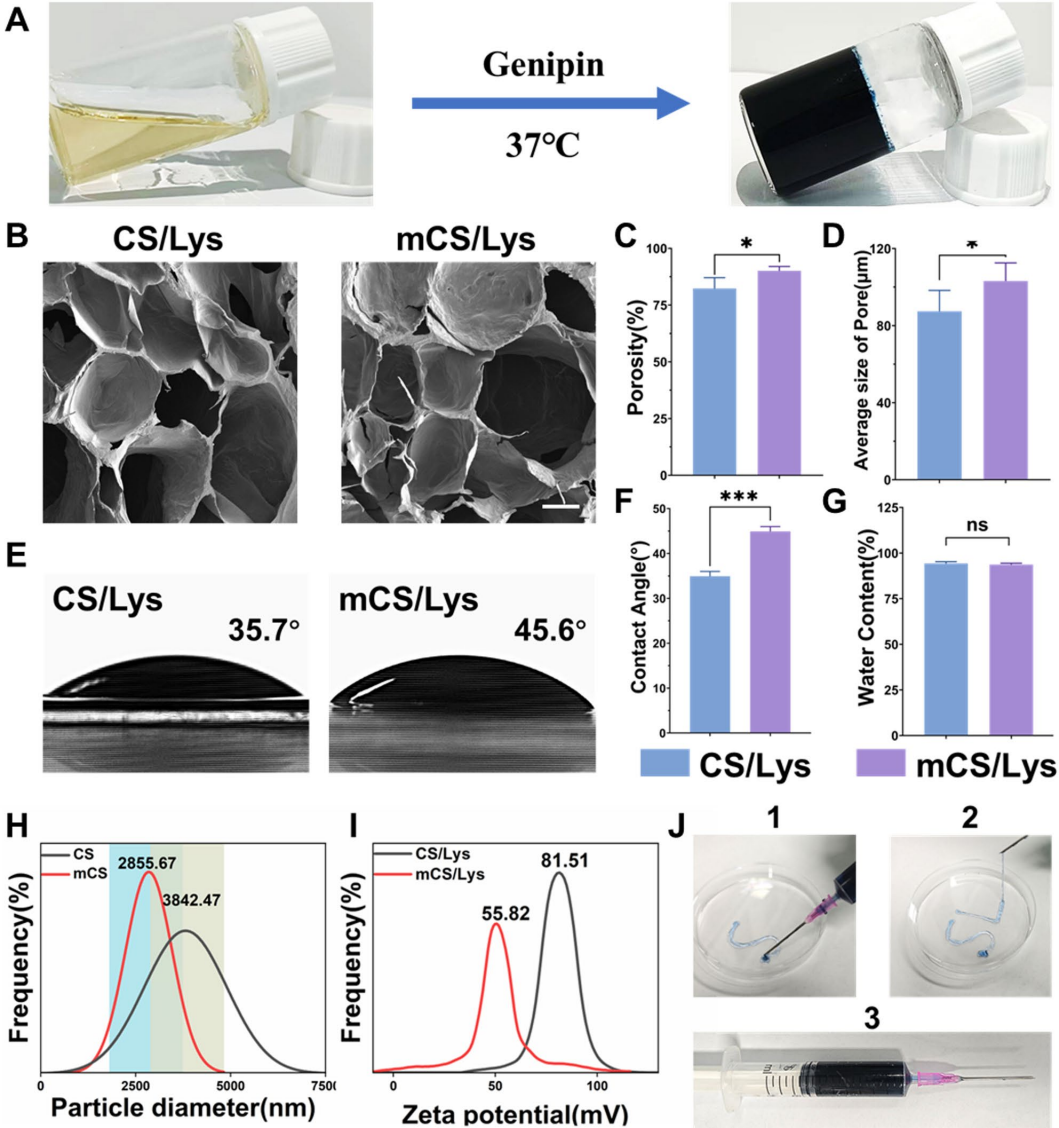
Physicochemical characterization of mCS/Lys hydrogels. **A**) photograph of the gelation process. **B-D**) Representative SEM images and quantitative analysis of CS/Lys hydrogels and mCS/Lys hydrogels (scale bar = 200 μm). **E-G**) Surface wettability assessments of CS/Lys hydrogels and mCS/Lys hydrogels. **F**) Analysis of water absorption kinetics for mCS/Lys hydrogels. **H**,** I**) Hydrodynamic diameter and zeta potentials of chitosan before and after alkylation, as determined by DLS and ELS. **J**) The injectability of mCS/Lys hydrogels. (*ns*: Not Significant, *: *p* < 0.05, ***: *p* < 0.001; mean ± SD, *n* = 5)

The successfully synthesized hydrogel presents as a sky-blue gel (Fig. 2A, S1). SEM revealed that the hydrogel possessed a well-defined three-dimensional (3D) porous architecture, closely resembling the fibrous mesh structure of native articular cartilage (Fig. 2B). Such a morphology is favorable for nutrient diffusion and extracellular matrix-like integration, highlighting its potential for cartilage tissue engineering applications [55]. Alkylation modification led to a slight increase in porosity from 82.3% to 90.1%, along with an expansion of the mean pore size from 87.5 ± 8.9 μm to 103.2 ± 7.7 μm (Fig. 2C–D). The relatively modest changes are likely attributable to the reduced extent of intermolecular hydrogen bonding and partial phase separation during gelation, which may have altered the packing density of the polymer chains [56]. Importantly, the retained high porosity and interconnected pore networks ensure sufficient space for cellular infiltration and matrix deposition, while mitigating excessive mechanical brittleness often associated with densely crosslinked hydrogels.

Contact angle measurements indicated that alkylation conferred enhanced surface hydrophobicity, with the water contact angle increasing from 34.9° to 44.9° (Fig. 2E–F). Despite this shift, the hydrogel remained moderately hydrophilic, a property reminiscent of native cartilage tissues, which typically exhibit contact angles in the range of 30–50°. This balance between hydrophilicity and hydrophobicity is advantageous for maintaining hydration while limiting protein denaturation or adverse cellular responses at the material interface. Water uptake studies demonstrated comparable swelling capacities for both alkylated (93.7%) and non-alkylated (94.3%) hydrogels (Fig. 2G), indicating that the introduction of dodecyl side chains did not compromise the material’s ability to retain water. Given that water content is a critical parameter for mimicking the hydrated microenvironment of cartilage, these findings suggest that alkylation preserves the hydrogel’s bio-mimetic hydration characteristics without adversely affecting its swelling behavior.

Collectively, the microstructural and physiochemical analyses demonstrate that the alkylated hydrogel retains key architectural and surface features akin to natural cartilage, including high porosity, interconnected pores, moderate hydrophilicity, and excellent water retention [56, 57]. These attributes position the material as a promising scaffold for cartilage regeneration, offering a biomimetic microenvironment conducive to cell viability, migration, and extracellular matrix production [35].

**Fig. 3:**
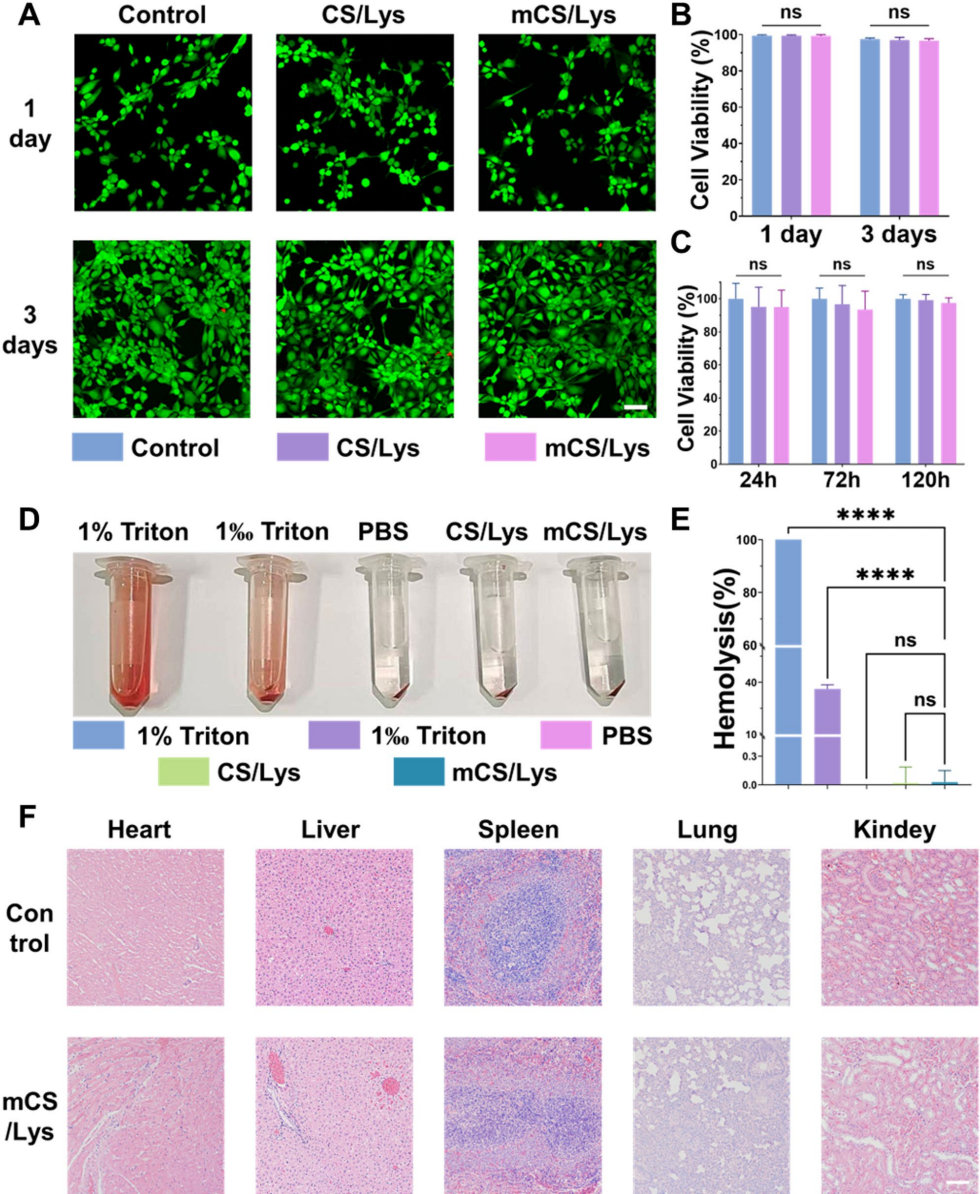
Assessment of the Biocompatibility of Hydrogels. **A**,** B**) Representative live/dead staining and corresponding quantification data of the gel co-cultured with ATDC5 cells (scale = 100 μm). **C**) Cell proliferation assay with ATDC5 cells. **D**,** E**) Hemolysis test of hydrogel extracts. **F**) H&E staining for tissue toxicity from hydrogels (scale bar = 100 μm). (*ns*: Not Significant, ***: *p* < 0.001; mean ± SD, *n* = 5)

Particle size and zeta potential measurements provided additional insight into the supramolecular assembly of the modified system: the hydrodynamic diameter decreased significantly from 3842.47 ± 26.28 nm to 2855.67 ± 20.11 nm post-modification(Fig. 2H). Due to alkylation modification, there was a reduction in free amino groups, resulting in the mCS/Lys group exhibiting a lower positive charge compared to the unmodified chitosan group, while still maintaining strong cationic properties (Fig. 2I, S3). Through electrostatic interactions, the hydrogel effectively binds to anionic cartilage and captures anionic nucleic acids, disrupting the metastable system of amorphous calcium phosphate (ACP) associated with nucleic acids, thereby inhibiting pathological calcification. Injectability assessments revealed smooth extrusion through a 26G needle, with “L”-shaped structures retaining their integrity (Fig. 2J 1–3). This property is attributed to the favorable viscoelastic properties and controllable gelation time of genipin hydrogels at moderate ratios under weakly acidic conditions, which facilitate intra-articular local injection and minimally invasive therapeutic applications [58].

Live/dead cell staining assay demonstrated a chondrocyte survival rate exceeding 95% after three days of exposure to 500 µg/mL hydrogel extracts, as evidenced by green fluorescence (Fig. 3A–B). The CCK-8 assay revealed no statistically significant difference in cell viability between the treated and control groups over a period of 120 h (Fig. 3C). Hemolysis assays indicated a rate of 2.8 ± 0.4%, which is substantially below the 5% threshold considered acceptable for biomaterials (Fig. 3D-E). In vivo safety assessment showed no signs of inflammation, necrosis, or structural damage in major organs after eight weeks (Fig. 3F). Collectively, these findings confirm that the mCS/Lys hydrogel exhibits excellent biocompatibility. Furthermore, results from intra-articular injection of the mCS/Lys hydrogel demonstrated its uniform distribution within cartilage (Fig. S4).

### Mechanical characterization of the cationic mCS/Lys hydrogel

**Fig. 4:**
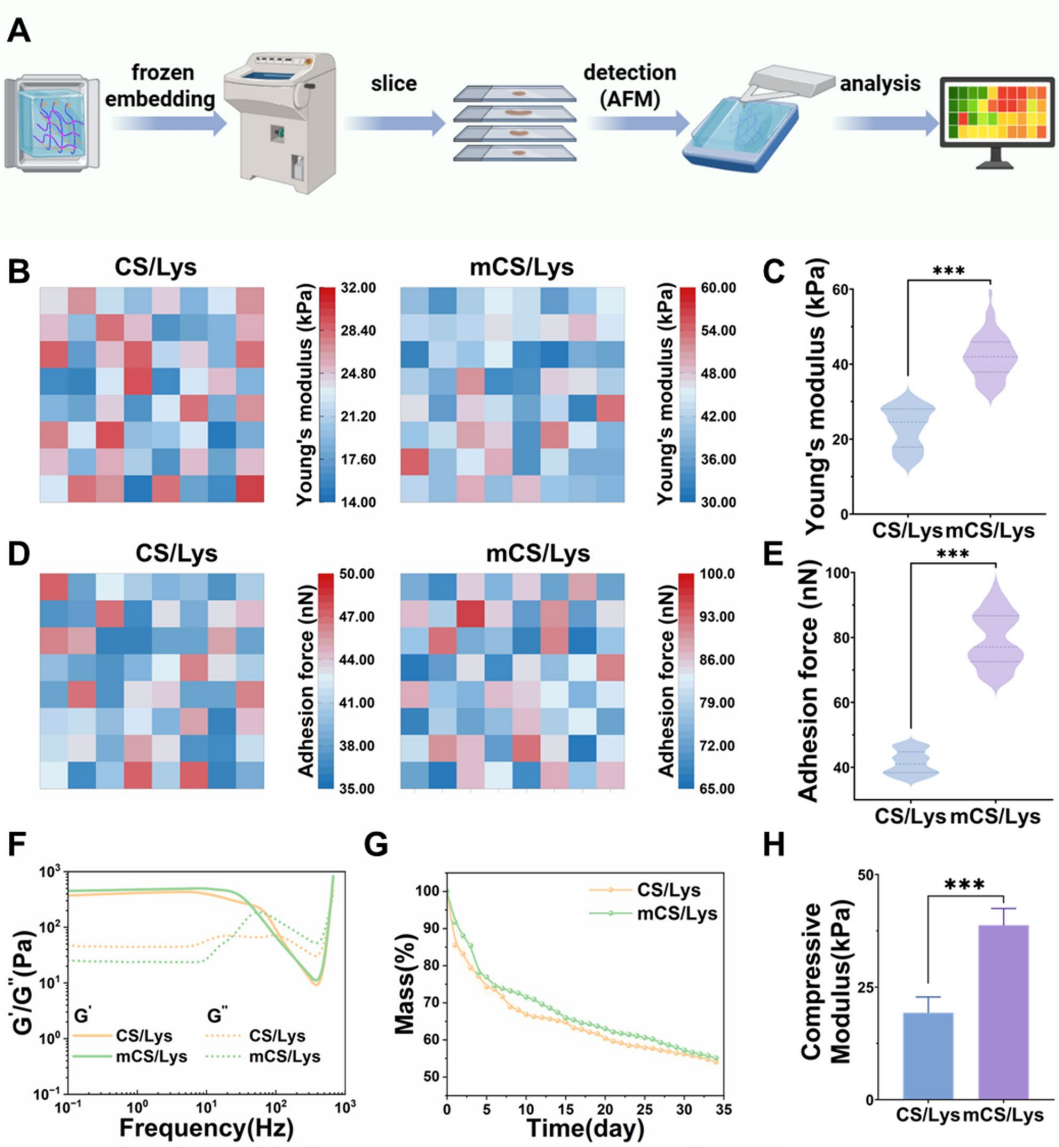
Mechanical Characterization of mCS/Lys Hydrogels. **A**) AFM detection process schematic for hydrogels. **B**,** C**) Heatmaps and statistical plots of Young’s modulus pre- and post-alkylation. **D**,** E**) Heatmaps and statistical plots of adhesion force pre- and post-alkylation. F) Rheological frequency sweep. **G**) In vitro degradation. **H**) Compressive modulus characterization. (***: *p* < 0.001; mean ± SD, *n* = 5)

The complex and dynamic loading environment of diarthrodial joints imposes stringent requirements on the mechanical performance of biomaterials designed for cartilage repair. To systematically evaluate the suitability of our cationic mCS/Lys hydrogel, we performed a comprehensive suite of mechanical and stability assessments. AFM nanoindentation revealed that alkylation significantly enhanced the local mechanical stiffness, with the Young’s modulus increasing from 23.1 ± 5.4 kPa to 42.1 ± 5.8 kPa, and the adhesion force rising from 41.4 ± 3.5 nN to 79.1 ± 8.1 nN (Fig. 4A–E). We attribute this improvement to three synergistic mechanisms: (i) physical interlocking of hydrophobic dodecyl chains, which elevates cohesive energy and reinforces interfacial interactions [59]; (ii) enhanced entropic elasticity due to restricted segmental mobility of polymer chains, thereby increasing resistance to deformation [60]; and (iii) additional covalent crosslinking via genipin-mediated bridging, which augments the overall network integrity [61].

Rheological analysis further corroborated the robust gel-like character of the alkylated hydrogel. The storage modulus (*G′*) reached 475.7 ± 16.2 Pa, nearly twentyfold higher than that of the loss modulus (*G”*)(24.2 ± 0.6 Pa), while the loss tangent (*tan δ*) remained below 0.1 across a broad frequency range of 0.1–100 Hz (Fig. 4F). This profile is indicative of a predominantly elastic solid response, essential for withstanding repetitive joint loading without irreversible deformation. Degradation resistance under physiological conditions is another critical parameter for implantable scaffolds. In enzymatic degradation tests conducted in PBS containing hyaluronidase at 37°C, the alkylated hydrogel retained 53.2% of its initial mass after 35 days, markedly superior to the rapid breakdown observed for the unmodified counterpart (Fig. 4G). This prolonged stability suggests that hydrophobic aggregation and enhanced crosslinking synergistically mitigate hydrolytic and enzymatic attack, thereby extending functional lifetime in vivo.

Compressive mechanical testing via bio-nano indenter yielded a compressive modulus of 38.7 ± 2.6 kPa for the modified hydrogel, more than double that of the control (19.3 ± 3.3 kPa) (Fig. 4H). Such augmentation in compressive stiffness directly translates to improved load-bearing capability, a prerequisite for matching the mechanical milieu of native articular cartilage [4]. Totally, these data demonstrate that alkylation confers substantial improvements in stiffness, adhesive interaction, elastic response, and environmental stability, while preserving the hydrogel’s water-rich, bio-mimetic character. The resulting mechanical profile positions the mCS/Lys hydrogel as a promising candidate for cartilage tissue engineering, where durable yet biocompatible scaffolds must simultaneously sustain physiologic stresses and support cellular activity.

### Lubrication performance and mechanism of the cationic mCS/Lys hydrogel

Current clinical strategies for OA lubrication therapy remain suboptimal. Intra-articular injection of HA provides only transient symptomatic relief and is associated with adverse effects such as acute pain and synovitis [4]. Moreover, rapid enzymatic degradation of HA in the joint necessitates frequent administration, thereby increasing the risk of infection and imposing a considerable burden on patients [10, 11]. Existing synthetic lubricants often fail to selectively target damaged cartilage surfaces [62] or to actively recruit endogenous lubricants (e.g., PC), resulting in insufficient lubrication under high load [63, 64].

**Fig. 5:**
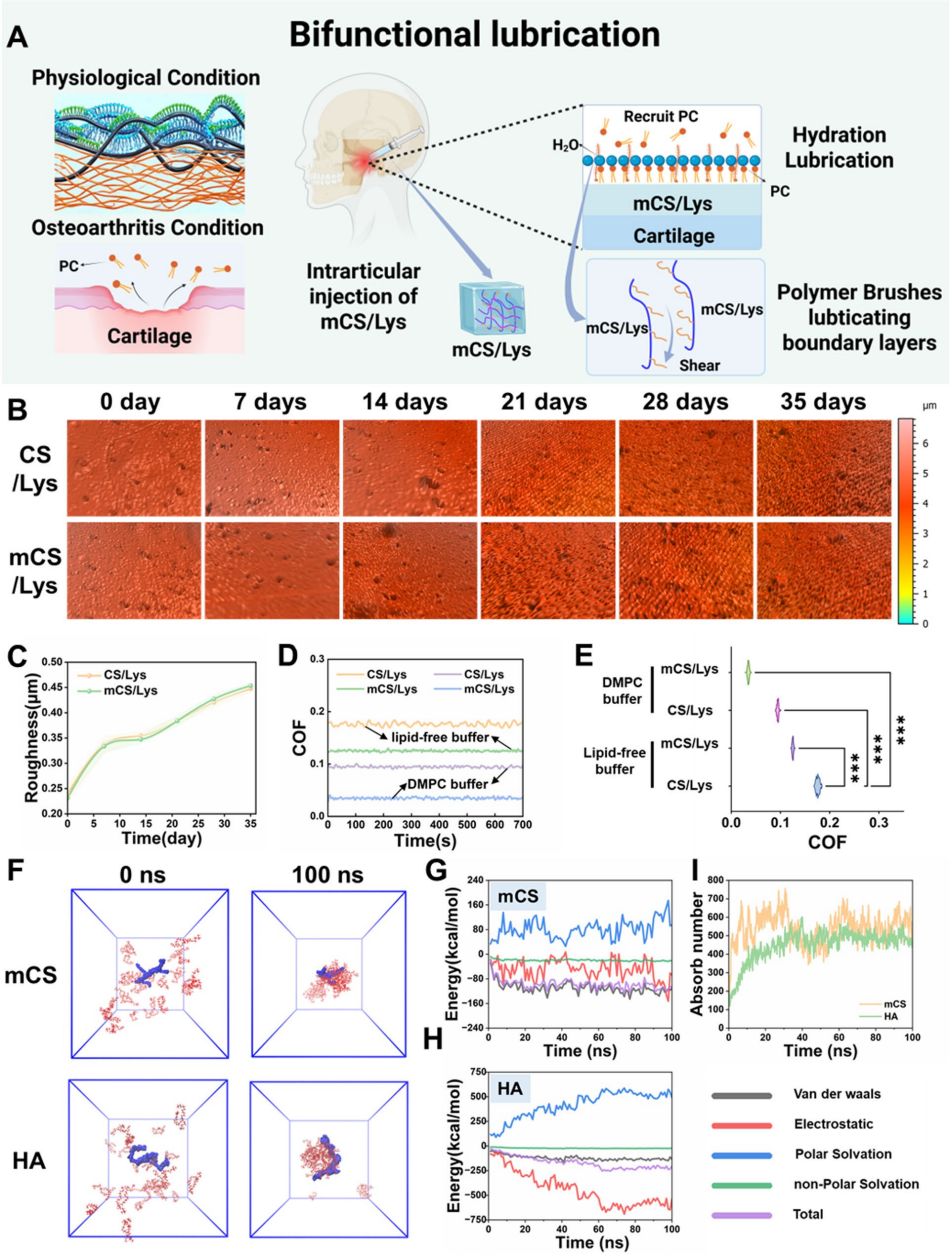
Lubrication performance of mCS/Lys hydrogels elucidated by molecular simulation. **A**) Schematic illustration of the in vivo lubrication mechanism. **B**,** C**) Time-dependent changes in surface roughness and quantitative analysis of the hydrogel. **D**, **E**) Evolution of COF over timefor mCS/Lys hydrogels. **F-I**) MD depicting the adsorption of mCS and hyaluronic acid onto a phosphatidylcholine membrane. (***: *p* < 0.001; mean ± SD, *n* = 5)

To address these limitations, we developed a cationic mCS/Lys hydrogel capable of delivering sustained low-friction performance through two complementary mechanisms (Fig. 5A). First, the hydrogel acts as a supramolecular reservoir to capture and retain PC from synovial fluid via electrostatic attraction and intermolecular interactions, thereby forming a stable boundary lubrication layer enriched with a hydrated interface [65]. Second, the grafted dodecyl side chains adopt a brush-like conformation that minimizes polymer chain interpenetration and suppresses entropic friction during sliding, effectively compensating for surface asperities and lubricant depletion characteristic of osteoarthritic joints [66].

Morphological stability under simulated physiological conditions is vital for continuous lubrication. Over 35 days in artificial synovial fluid, the mCS/Lys hydrogel maintained a low surface roughness (Ra = 453.5 ± 4.9 nm), comparable to that of unmodified CS/Lys (Ra = 447.5 ± 7.4 nm), reflecting robust interfacial integrity even in the presence of enzymatic and mechanical challenges (Fig. 5B, C, and S5). Under physiological contact pressures (maximum 25.68 MPa, normal load 1 N) [67, 68], the mCS/Lys hydrogel exhibited a markedly reduced COF of 0.125 ± 0.006 versus 0.176 ± 0.008 for CS/Lys (Fig. 5D, E). Upon supplementation with 1 mM exogenous PC, the COF of mCS/Lys further decreased to 0.036 ± 0.003, whereas CS/Lys achieved only 0.095 ± 0.020. This pronounced enhancement underscores the pivotal role of PC adsorption in establishing a hydrated lubricating film that resists shear-induced failure.

MD simulations provided atomistic insight into the supramolecular recognition between mCS and PC. The calculated non-covalent binding energy was − 97.3 kcal·mol⁻¹, and the equilibrium adsorption plateaued at 0.46 ± 0.03 PC molecules per mCS monomer over a 100 ns trajectory (Fig. 5F–I). Notably, these values are comparable to those reported for HA (0.41 ± 0.02 PC per disaccharide unit), confirming that the mCS/Lys system possesses a PC-binding affinity on par with natural synovial lubricants. Taken together, by integrating “supramolecular PC capture” with “entropy-driven friction reduction via alkyl side chains,” the mCS/Lys hydrogel delivers durable low-friction protection, mitigates cartilage wear, and prolongs intra-articular residence time, thereby reducing the frequency of clinical intervention. This dual-functional design not only addresses the biomechanical shortcomings of current lubricants but also aligns with the biochemical landscape of the degenerated joint, offering a promising translational avenue for OA therapy.

### Therapeutic efficacy of the cationic mCS/Lys hydrogel in a temporomandibular joint osteoarthritis model

**Fig. 6:**
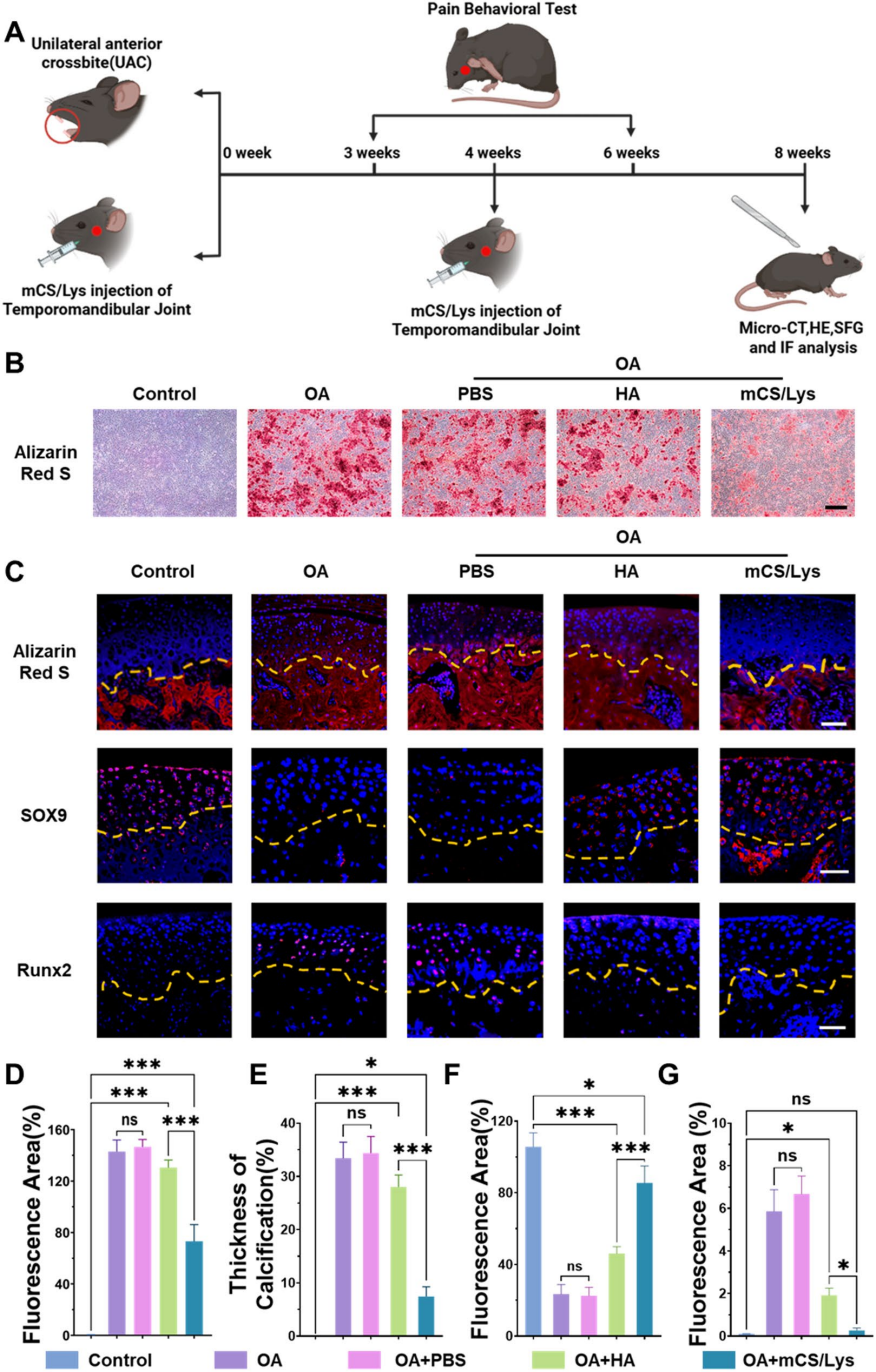
Mitigation of pathological calcification by mCS/Lys hydrogels. **A**) Schematic of the experimental protocol for the in vivo animal model. **B**) Representative alizarin red staining images of in vitro samples (scale bar = 200 μm). **C**) In vivo alizarin red staining and immunofluorescence imaging of calcification-associated proteins (scale bars = 30 μm). **D**) Semi-quantitative analysis of in vitro mineralization. **E-G**) Semi-quantitative analysis of in vivo calcification markers. (*ns*: Not Significant, *: *p* < 0.05, ***: *p* < 0.001; mean ± SD, *n* = 5)

To evaluate the in vivo therapeutic potential of the cationic mCS/Lys hydrogel, we employed a murine UAC model that recapitulates key features of TMJOA [26, 44]. Mice received intra-articular injections of mCS/Lys, HA, or PBS every four weeks, with endpoint analyses conducted at eight weeks (Fig. 6A). In vitro alizarin red staining revealed that the OA + mCS/Lys group exhibited a 48.8 ± 8.1% reduction in calcified nodule area relative to the OA control, outperforming the OA + HA group, which showed only an 8.7 ± 3.7% decrease (Fig. 6B, D). Complementary in vivo quantification demonstrated that mCS/Lys lowered the ratio of calcified cartilage to total cartilage thickness by 78.4 ± 4.3%, significantly greater than the 18.5 ± 5.3% reduction achieved with HA (Fig. 6C, E). These findings establish mCS/Lys as a more potent suppressor of pathological calcification than the current clinical standard.

Immunofluorescence analysis further delineated the molecular basis for enhanced cartilage regeneration. Expression of SOX9, a master transcription factor driving chondrogenic matrix fabrication, was elevated 40.1-fold in the OA + mCS/Lys group—more than twice the 19.5-fold induction seen with HA (Fig. 6C, F). Conversely, RUNX2, a pivotal mediator of osteogenic differentiation, was downregulated by 95.5% in OA + mCS/Lys animals, compared with a 67.4% reduction in the OA + HA cohort (Fig. 6C, G). Collectively, these data highlight the superior capacity of mCS/Lys to promote chondroprotective gene programs while concurrently inhibiting aberrant osteogenesis.

**Fig. 7:**
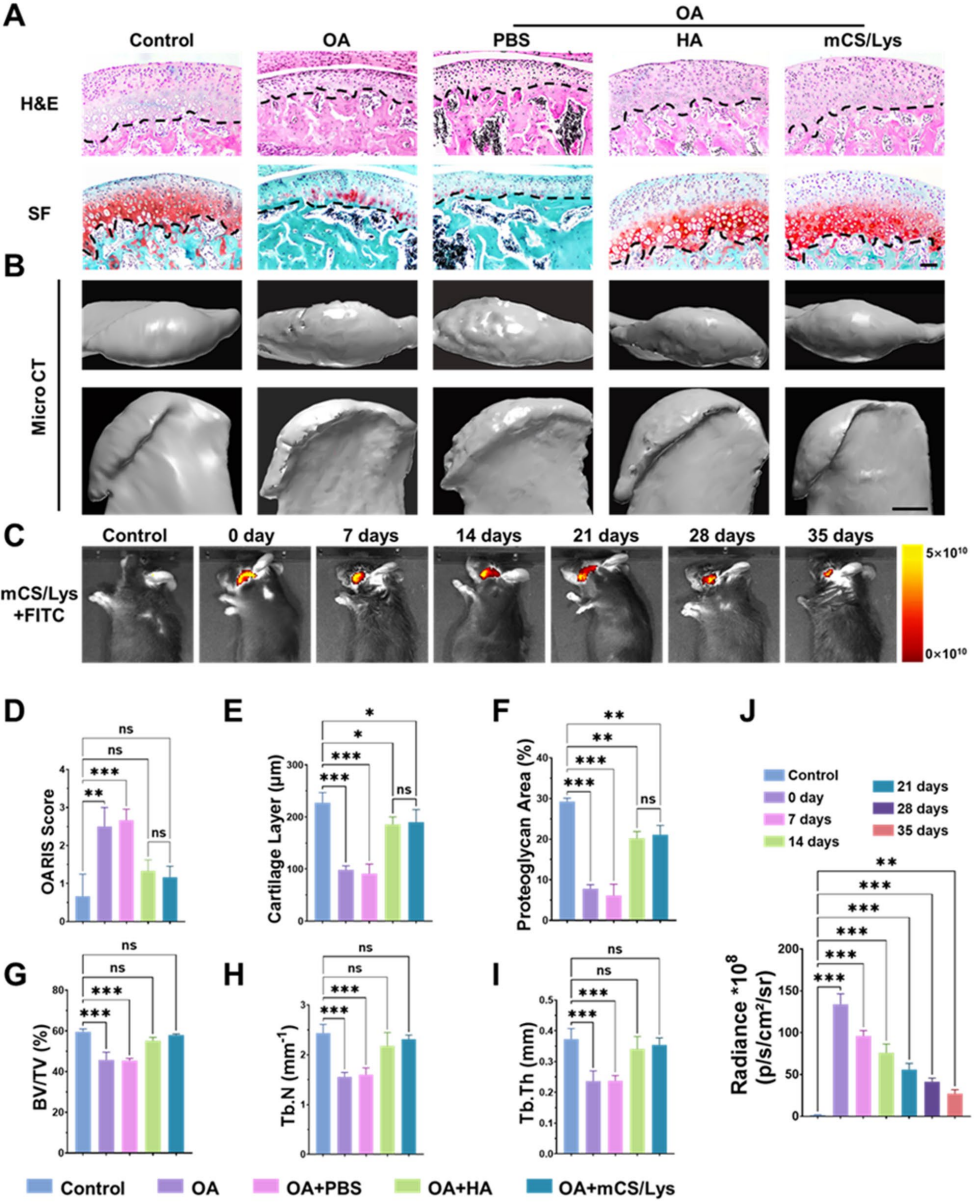
In vivo persistence and therapeutic efficacy of mCS/Lys hydrogels in a mouse model of TMJOA. **A**) Representative histological sections stained with H&E and SF (scale bar = 50 μm). **B**) Representative Micro-CT images of subchondral bone (scale bar = 250 μm). **C**) In vivo fluorescence imaging tracking the residence of FITC-labeled mCS/Lys hydrogel post-injection. **D-F**) Semiquantitative analysis of OARSI histopathology scores and SF staining intensity. **G-I**) Micro-CT-based quantitative assessment of bone volume/total volume (BV/TV), trabecular thickness (Tb.Th), and trabecular number (Tb.N). **J**) Longitudinal in vivo bioluminescence imaging monitoring hydrogel persistence. (*ns*: Not Significant, *: *p* < 0.05, **: *p* < 0.01, ***: *p* < 0.001; mean ± SD, *n* = 5)

Histological analysis confirmed the molecular findings. SF and H&E staining revealed that the OA + mCS/Lys group maintained intact cartilage with well-organized chondrocytes, unlike the OA + PBS group, which showed severe matrix loss and disintegration (Fig. 7A). Quantitative scores supported these observations, with lower OARSI scores in OA + mCS/Lys (1.2 ± 0.2) and OA + HA (1.3 ± 0.3) compared to OA + PBS (2.7 ± 0.2), indicating reduced cartilage degeneration. Treated groups also had greater cartilage thickness (OA + mCS/Lys: 189.8 ± 21.8 μm; OA + HA: 185.9 ± 12.6 μm) than controls (91.3 ± 16.4 μm), and better proteoglycan distribution (OA + mCS/Lys: 21.1 ± 1.9%; OA + HA: 20.2 ± 1.4%) compared to PBS (6.2 ± 2.2%) (Fig. 7D–F).

MicroCT analysis showed that mCS/Lys not only repaired articular cartilage but also restored subchondral bone architecture (Fig. 7B, G–I). The bone volume fraction (BV/TV) was significantly higher in the OA + mCS/Lys (57.91 ± 0.49%) and OA + HA (55.22 ± 1.31%) groups compared to OA + PBS (45.46 ± 0.84%), along with increased trabecular number and thickness. These findings suggest that mCS/Lys promotes simultaneous regeneration of cartilage and bone, addressing the complex pathology of OA. Long-term retention is essential for effective treatment. In vivo fluorescence imaging showed that FITC-labeled mCS/Lys retained 20.3 ± 1.8% of its initial signal in the TMJ cavity at day 35, significantly higher than the control and surpassing the 14-day retention of HA (Fig. 7C, J, and Fig. S6–7). This extended presence ensures ongoing lubrication and anti-calcification in the degenerative joint environment.

Unlike traditional OA treatments, HA doesn’t prevent calcification, and current inhibitors don’t provide lubrication. The mCS/Lys system combines both functions: it provides boundary lubrication via brush-like alkyl chains that enhance PC adsorption and prevent calcification by chelating calcium and aggregating nucleic acids. Its injectable form and excellent rheology ensure even distribution in irregular osteochondral defects, enhancing therapeutic coverage. This dual-function approach results in better material retention and outcomes than single treatments, providing a versatile OA management platform.

### Systematic investigation into the analgesic effects of cationic mCS/Lys hydrogel

**Fig. 8:**
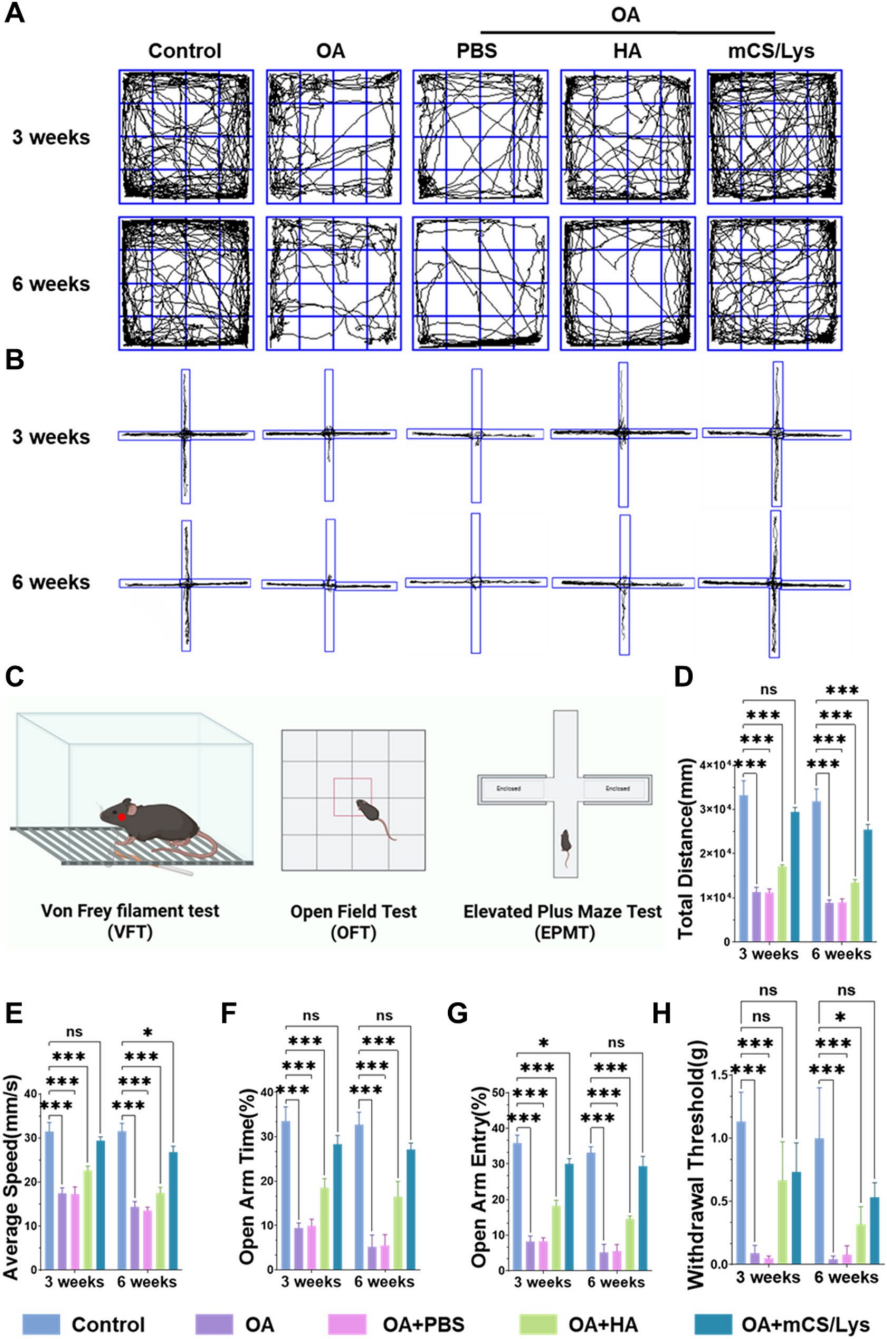
Assessment of pain-related behaviors in mCS/Lys hydrogel-treated TMJOA mice. **A**,** B**) Representative locomotion tracks in OFT and EPM at 3 and 6 weeks post-modeling. **C**) Schematic diagram of the behavioral testing protocols. **D-G**) Quantitative analysis of total travel distance in the OFT and time spent in the open arms of the EPM. **H**) Mechanical allodynia assessed by the von Frey filament test. (*ns*: Not Significant, *: *p* < 0.05, ***: *p* < 0.001; mean ± SD, *n* = 5)

Pain represents the predominant symptom of OA and severely compromises patients’ quality of life [69, 70]. Consequently, evaluating the analgesic potential of therapeutic hydrogels is essential for meaningful OA management. To comprehensively assess pain-related behaviors, we performed multi-dimensional behavioral tests, including assessments of anxiety-like responses and mechanical hyperalgesia (Fig. 8C).

Anxiety levels were quantified using EPM and OFT. Both the OA + mCS/Lys and OA + HA groups exhibited significantly lower anxiety-related indices than the OA + PBS control, with mCS/Lys demonstrating superior anxiolytic effects relative to HA. In the OFT, the OA + mCS/Lys group covered a greater total distance (29 433.8 ± 2943.8 mm at 3 weeks; 25 468.6 ± 930.9 mm at 6 weeks) and maintained a higher average locomotor speed (29.4 ± 0.7 mm/s at 3 weeks; 26.8 ± 1.1 mm/s at 6 weeks) than both HA-treated and PBS-treated cohorts (Fig. 8A, D–E). Similarly, EPM analysis revealed that the OA + mCS/Lys group spent a markedly higher percentage of time in the open arms (28.4 ± 1.6% at 3 weeks; 27.1 ± 1.2% at 6 weeks) and displayed a higher frequency of open-arm entries (30.0 ± 1.2% at 3 weeks; 29.4 ± 2.2% at 6 weeks) compared to OA + HA and OA + PBS groups (Fig. 8B, F–G). These findings indicate enhanced exploratory behavior and reduced anxiety-like phenotypes, implying that mCS/Lys more effectively alleviates OA-associated spontaneous pain-induced anxiety than HA.

Mechanical hyperalgesia was evaluated via the von Frey filament assay (Fig. 8H). The OA + mCS/Lys group exhibited progressive pain threshold recovery, reaching 61.7 ± 18.1% at 3 weeks and 51.2 ± 9.8% at 6 weeks—both exceeding the predefined 50% effective relief benchmark. By contrast, the OA + HA group achieved 55.3 ± 13.9% recovery at 3 weeks, which declined to 28.9 ± 11.8% at 6 weeks. This temporal profile underscores the sustained analgesic effect of mCS/Lys, which parallels HA in the short term but surpasses it over longer periods, likely attributable to the hydrogel’s prolonged intra-articular retention (Fig. 7C). Collectively, mCS/Lys hydrogel confers superior mitigation of OA-induced anxiety and spontaneous pain, and provides extended relief from mechanical hyperalgesia, positioning it as a promising candidate for comprehensive TMJOA management.

Mechanistically, the observed analgesia stems from two interrelated processes. First, boundary lubrication afforded by the hydrogel reduces frictional forces, thereby diminishing abnormal mechanical loading on nociceptors embedded in subchondral bone and synovial tissues [70]. Second, cartilage repair elicits neuroimmune modulation by restoring extracellular matrix integrity and attenuating pro-inflammatory nociceptive signaling [69]. Although the immediate pain reduction with mCS/Lys mirrors that of HA, the durability of analgesia in the mCS/Lys cohort reflects ongoing tissue regeneration and a corresponding decline in persistent nociceptive input. These insights highlight the need for future investigations into the specific mechanotransduction and immunomodulatory pathways linking material-driven repair to long-term pain relief.

### Therapeutic mechanism of cationic mCS/Lys hydrogel

Extracellular nucleic acids (both DNA and RNA) are emerging as pivotal mediators of pathological calcification in OA [7]. Their polyanionic nature enables stabilization of ACP precursors, fostering the formation of nucleic acid–ACP nanocomplexes that impede crystal phase transitions. These nanocomplexes adsorb onto collagen fibrils via hydrogen bonding and van der Waals interactions, facilitating ACP infiltration into the fibril core and promoting intrafibrillar mineralization [71]. At the molecular level, extracellular nucleic acids activate the VEGFR2 signaling cascade, enhancing neurovascular ingrowth and modulating cellular autophagy as well as osteogenic gene expression [46]. Importantly, nucleic acid deposition spatially colocalizes with calcific foci, and targeted degradation of these species can reverse the calcification process.

**Fig. 9:**
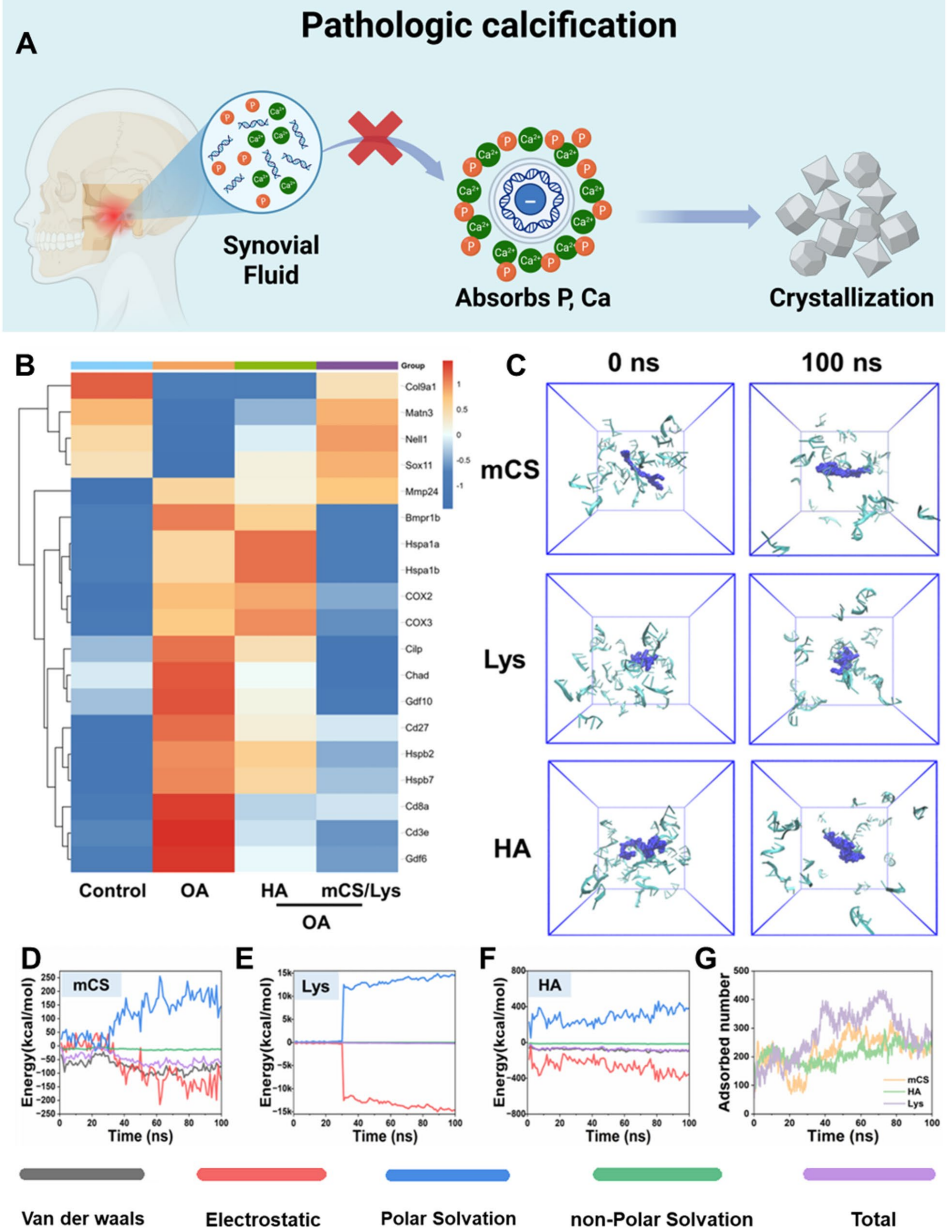
Proposed mechanism by which mCS/Lys hydrogels inhibit pathological calcification. **A**) Schematic illustration of mCS/Lys sequestering extracellular nucleic acids to impede calcification initiation. **B**) Heatmap displaying alterations in the expression of key cartilage anabolic and catabolic genes under different treatments. **C**) Snapshots from molecular dynamics simulations showing nucleic acid adsorption onto mCS, Lys, and HA surfaces at 0 ns and 100 ns. **D-F**) Bar charts of the calculated binding energies for each component. **G**) Temporal evolution of adsorbed atomic species on the surface of mCS, Lys, and HA

Building on this pathological framework, the cationic mCS/Lys hydrogel counteracts calcification through a multifaceted mechanism (Fig. 9A). RNA sequencing (RNA-seq) analysis identified 286 differentially expressed genes implicated in major OA pathways (SOX, MMP, COX, HSPA, BMP, CILP) (Fig. 9B). Both mCS/Lys and HA ameliorated TMJOA pathology; however, mCS/Lys preferentially upregulated cartilage-protective genes, particularly those of the SOX and COL families, consistent with its superior immunofluorescence findings of SOX9 elevation and RUNX2 suppression. In contrast, HA elicited a marked induction of pain-related genes such as COX2, aligning with the behavioral assessments in Sect.  3.5, which revealed diminished long-term analgesia compared to mCS/Lys. These transcriptomic signatures suggest that mCS/Lys stabilizes the chondrocytic phenotype and attenuates calcification by orchestrating gene expression programs favoring matrix preservation over osteogenic differentiation.

MD simulations provided atomistic insight into the nucleic acid–binding capabilities of mCS/Lys constituents. Alkylated chitosan and lysine exhibited binding energies of − 66.15 kcal·mol⁻¹ and − 148.9 kcal·mol⁻¹, respectively, toward extracellular nucleic acids—comparable to, yet in the case of lysine, exceeding that of HA (–77.9 kcal·mol⁻¹) (Fig. 9C–G). Experimental adsorption assays corroborated these computational predictions: mCS and Lys bound 1.95 ± 0.51 and 2.49 ± 0.74 nucleic acid molecules per monomer, respectively, outperforming HA (1.86 ± 0.28) (Fig. 9C–G). Together, these data demonstrate that mCS/Lys possesses a superior capacity to sequester extracellular nucleic acids, thereby disrupting the initial steps of calcific nodule formation.

The mCS/Lys hydrogel chelates extracellular nucleic acids, causing flocculation, inhibiting hydroxyapatite crystallization, and modulating cartilage matrix gene expression. This dual-action approach prevents calcification and maintains extracellular matrix integrity, offering a long-lasting anti-calcification solution unlike existing single-function treatments. This study uniquely targets the interconnected pathological loop in OA progression, a gap ignored by therapies focusing on isolated factors. Our findings show that simply restoring lubrication isn’t enough to stop OA, as ongoing calcific damage disrupts joint stability, making dual functionalization essential. The prolonged intra-articular presence of mCS/Lys surpasses the short-lived effects of HA and many lab-engineered agents, reducing the need for frequent injections.

This approach reduces the burdens and risks of repeated joint penetrations. The rheological properties and injectability of mCS/Lys enable even distribution in irregular cartilage defects, enhancing therapeutic coverage. The mCS/Lys hydrogel successfully breaks the cycle of lubrication failure and calcific degeneration. This dual-function strategy offers a more comprehensive and lasting solution than conventional treatments by addressing both main drivers of OA. It prevents calcification, maintains matrix integrity, and provides long-lasting lubrication, overcoming current therapy limitations. This work sets the stage for next-generation OA treatments by demonstrating the effectiveness of dual-functionality in targeting interconnected disease mechanisms. This study focuses on TMJOA, a small joint with unique properties, limiting the findings’ applicability to larger joints like the knee, which have different mechanisms and stresses. Future research should systematically explore large-joint models to address these gaps and advance joint-agnostic OA therapies.

## Conclusion

We have developed an injectable, dual-functional cationic mCS/Lys hydrogel designed to address the root cause of OA by disrupting the pathogenic lubrication–calcification coupling. This hydrogel employs a brush-like molecular architecture and endogenous lubricant adsorption to provide sustained lubrication and effectively suppress nucleic acid-driven pathological calcification through electrostatic binding, thereby outperforming clinical hyaluronic acid. In a murine TMJOA model, the hydrogel significantly reduced cartilage degeneration, delayed disease progression, and alleviated chronic pain. By combining prolonged lubrication with anti-calcification properties, our approach transitions OA therapy from symptomatic management to source-level intervention, potentially signaling a paradigm shift in treatment. Future work will focus on optimizing the material synthesis process and modification ratio, while also employing additional models, such as animal models of knee osteoarthritis, to verify the broad applicability of the hydrogel.

## Supplementary Information

Supplementary Material 1.

## Data Availability

No datasets were generated or analysed during the current study.

## Abbreviations

mCS: Alkylated modified chitosan
ACP: Amorphous calcium phosphate
ARRIVE guidelines: Animal Research: Reporting of In Vivo Experiments
AFM: Atomic force microscopy
ATR-FTIR: attenuated total reflectance-Fourier transform infrared spectroscopy
CCK-8: cell counting kit-8
CS: chitosa
COF: coefficient of friction
DLS: Dynamic light scattering
ELS: electrophoretic light scattering
EPM: elevated plus maze
exNA: extracellular nucleic acids
FBS: fetal bovine serum
FITC: fluorescein isothiocyanate
HA: hyaluronic acid
Lys: lysine
αMEM: minimum essential mediumα
MD: molecular dynamics
NEJM: New England Journal of Medicine
OFT: open field test
OA: osteoarthritis
OARSI: osteoarthritis research society international
PBS: phosphate buffer saline
PC: phosphatidylcholine
SEM: scanning electron microscopy
SD: standard deviation
TMJOA: temporomandibular joint osteoarthritis
TGA: Thermogravimetric analysis
3D: three-dimensional
UAC: unilateral anterior crossbite
XRD: X-ray diffraction
