# Supplementary material for: Cationic hydrogel with sustained lubrication and anti-calcification for osteoarthritis therapy
Source: J Nanobiotechnology. 2026 Mar 1;24:316. doi: 10.1186/s12951-026-04164-0 (PMC13059275; doi:10.1186/s12951-026-04164-0)
Supplement: Supplementary file 1 — Supplementary Material 1. [file 12951_2026_4164_MOESM1_ESM.docx]

Supporting Information

**Cationic mCS/Lys Hydrogel with Sustained Lubrication and Anti-Calcification for Osteoarthritis Therapy**

**This SI file includes:**

Figure S1. Photographs of the gelation process of alkyl-modified chitosan hydrogels with different alkyl modification ratios

Figure S2. The FTIR results of the raw materials of mCS/Lys.

Figure S3. Zeta potential of non-modified chitosan and alkyl-modified chitosan hydrogels with different alkyl modification ratios (alkyl: chitosan = 10:1, 10:3, 10:5), and the solvent acetic acid.

Figure S4. The distribution of the FITC-stained hydrogel in condyles 14 weeks after injection.

Figure S5. 2D Images of surface roughness and quantitative analysis of non-alkyl-modified and alkyl-modified hydrogels as a function of time.

Figure S6. In vivo imaging results after intra-articular injection of hyaluronic acid–FITC into the temporomandibular joint cavity (Day 0).

Figure S7. In vivo imaging results after intra-articular injection of hyaluronic acid–FITC into the temporomandibular joint cavity (Day 14).


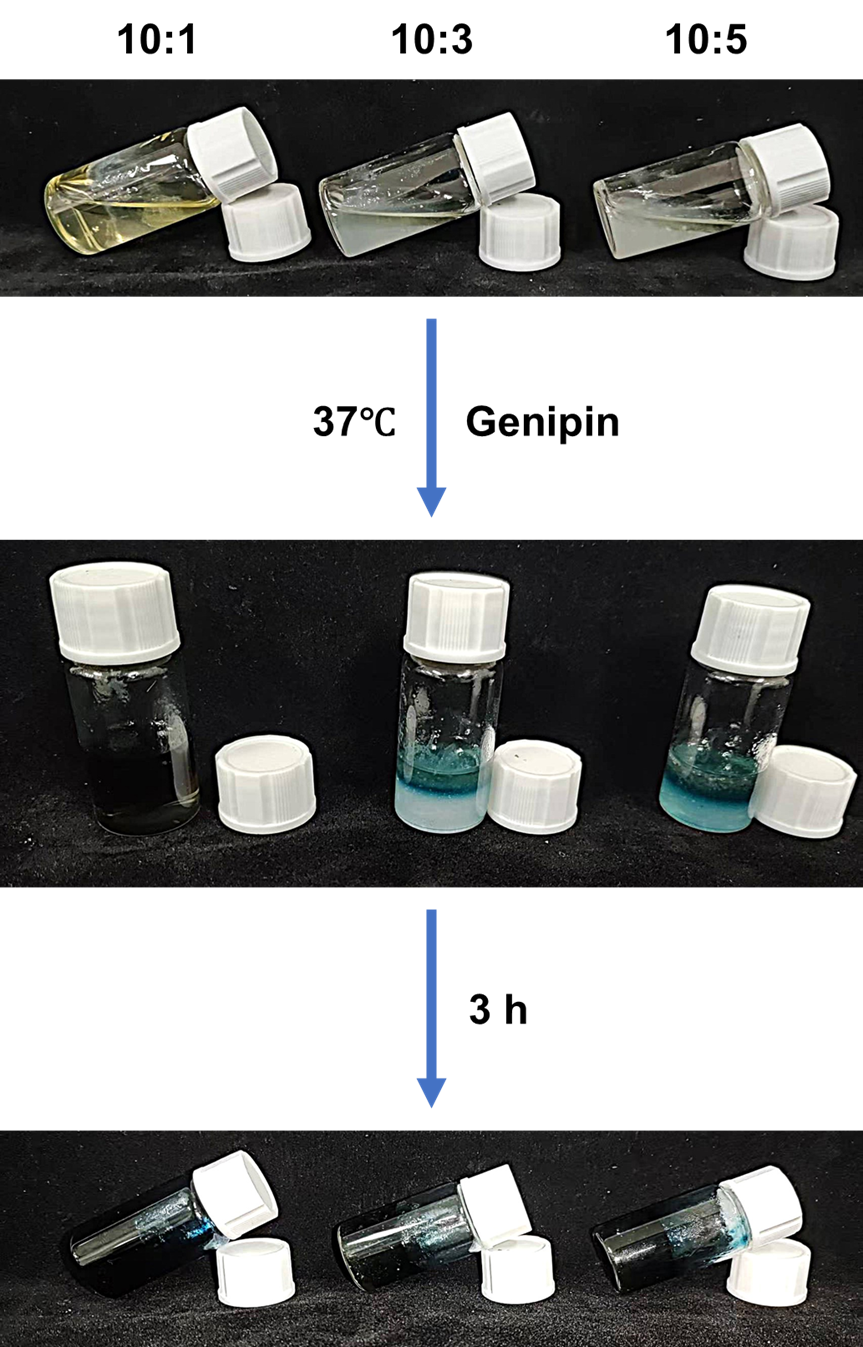


**Fig. S1.** Photographs of the gelation process of alkyl-modified chitosan hydrogels with different alkyl modification ratios (alkyl: chitosan = 10:1, 10:3, 10:5).


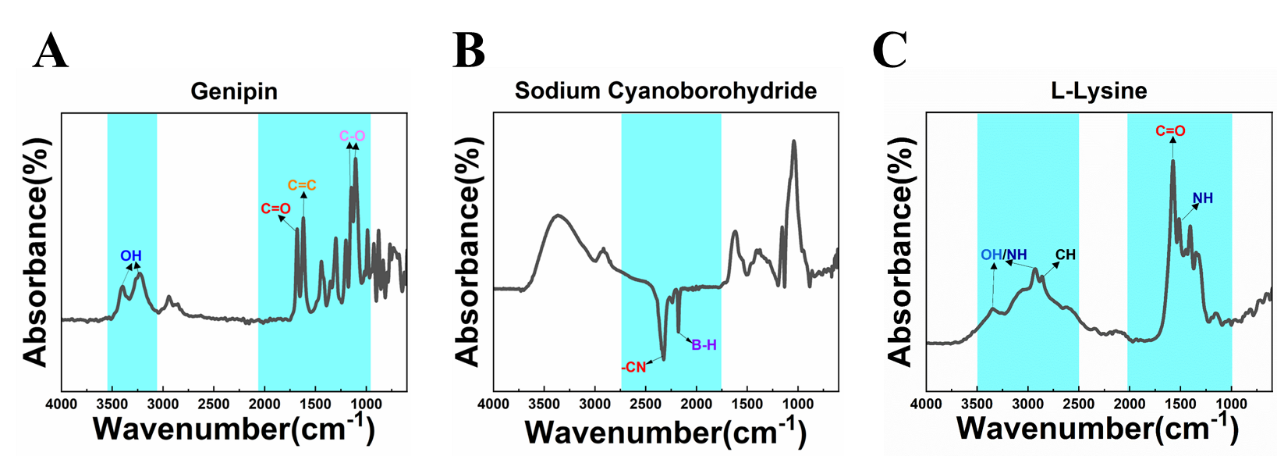


**Fig. S2.** The FTIR results of the raw materials of mCS/Lys. A) Genipin, B) Sodium Cyanoborohydride, C) L-Lysine (Lys).





**Fig. S3.** Zeta potential of non-modified chitosan and alkyl-modified chitosan hydrogels with different alkyl modification ratios (alkyl: chitosan = 10:1, 10:3, 10:5), and the solvent acetic acid.


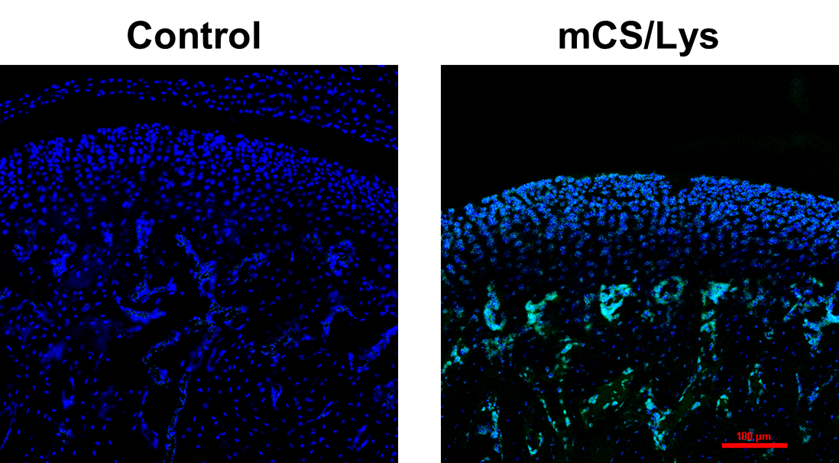


**Fig. S4.** The distribution of the FITC-stained hydrogel in condyles 4 weeks after injection. Scale bar = 100 μm.


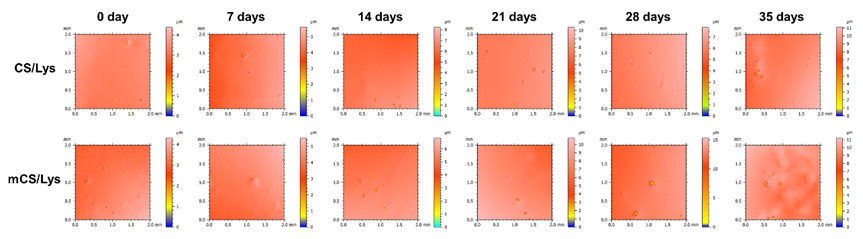


**Fig. S5.** 2D Images of non-alkyl-modified and alkyl-modified hydrogels as a function of time.


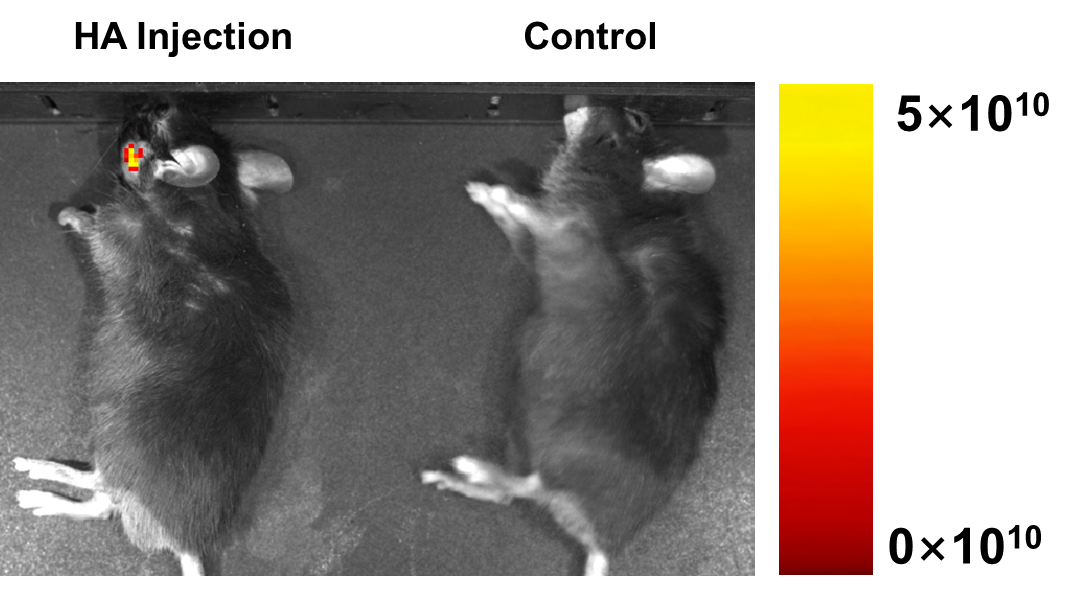


**Figure S6.** In vivo imaging results after intra-articular injection of hyaluronic acid–FITC into the temporomandibular joint cavity (Day 0).


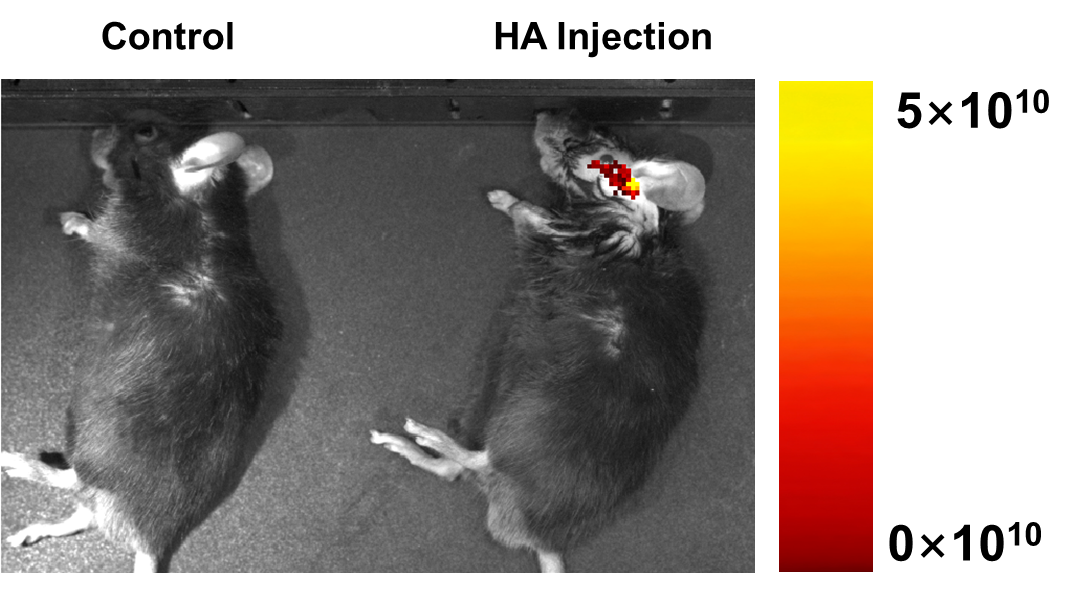


**Fig. S7.** In vivo imaging results after intra-articular injection of hyaluronic acid–FITC into the temporomandibular joint cavity (Day 14).
